# Antimicrobial Resistance and Clinical Outcomes of Uropathogenic Enterococcal Infections in Hospitalised Patients

**DOI:** 10.7759/cureus.108614

**Published:** 2026-05-10

**Authors:** Sujata P Korochikar, Priyanka M Mane, Satish R Patil, Yogendra P Shelke, Suvarna N Patil, Premkumar V Korochikar

**Affiliations:** 1 Department of Microbiology, Krishna Institute of Medical Sciences, Krishna Vishwa Vidyapeeth (Deemed to be University), Karad, IND; 2 Department of Microbiology, B.K.L. Walawalkar Rural Medical College, Chiplun, IND; 3 Department of Medicine, B.K.L. Walawalkar Rural Medical College, Chiplun, IND; 4 Department of Advanced Diploma in Medical Laboratory Technology (ADMLT), Shri Vithalrao Joshi Charities Trust’s College of Advanced Studies, Chiplun, IND

**Keywords:** antimicrobial resistance index (ari), clinical outcome, enterococcus faecalis, enterococcus faecium, multiple antibiotic resistance index (mari), urinary tract infection

## Abstract

Introduction

*Enterococcus faecalis* and *Enterococcus faecium* are important opportunistic pathogens causing urinary tract infections (UTIs) and are increasingly associated with antimicrobial resistance (AMR). This study aimed to evaluate the AMR patterns and clinical outcomes of uropathogenic *Enterococcus* species in hospitalised patients and to correlate resistance profiles with demographic and clinical parameters.

Methods

This prospective observational study was conducted at a tertiary care hospital in Southwest Maharashtra from October 2023 to October 2025. Thirty *Enterococcal *urinary isolates from hospitalised patients were analysed using standard microbiological techniques in accordance with the Clinical and Laboratory Standards Institute (CLSI) M100 guidelines. Parameters such as the Antimicrobial Resistance Index (ARI), the Multiple Antibiotic Resistance Index (MARI), and resistance categories were assessed in relation to clinical factors.

Results

*Enterococcus species* isolates accounted for 30/490 (6.12%) of the inpatient culture-positive urine specimens, with 14 (46.7%) *E. faecalis *and 16 (53.3%) *E. faecium*. *E. faecium *showed 16 (100%) resistance to antimicrobials such as β-lactams, fluoroquinolones, fosfomycin, and high-level streptomycin (HLS), whereas *E. faecalis *showed comparatively lower resistance, including three (21.4%) to β-lactams. Both species were susceptible to vancomycin and linezolid. Multidrug-resistant (MDR), extensively drug-resistant (XDR), and pandrug-resistant (PDR) phenotypes were more common in *E. faecium *than in *E. faecalis*. XDR isolates had the longest hospital stays, whereas non-MDR *E. faecalis* had the shortest. Complicated UTIs (cUTIs) were the most common type. Overall, 23 (76.7%) patients showed improvement, 3 (10%) left against medical advice (LAMA), and 4 (13.3%) died due to renal disease and geriatric comorbidities in MDR/XDR infections.

Conclusion

*E. faecium *showed comparatively higher resistance than *E. faecalis*, necessitating species-specific antimicrobial treatment. Targeted vigilance is required, especially for high-risk groups with device-related infections, uropathy and obstetric conditions. Antimicrobial stewardship and surveillance are essential for improving patient outcomes.

## Introduction

Urinary tract infections (UTIs) are a substantial public health concern in India, particularly in hospital settings, where *Enterococcus* species, notably *Enterococcus faecium *(*E. faecium*) and *Enterococcus faecalis* (*E. faecalis*), are commonly identified as significant uropathogens. The increasing prevalence of antimicrobial resistance (AMR) in *Enterococcus *species complicates patient treatment. This trend reflects global patterns, as meta-analyses have highlighted increased resistance to key antibacterial classes, including β-lactams, aminoglycosides, glycopeptides and fluoroquinolones. Resistance rates to several commonly used antibiotics have exceeded 30-40% among urinary isolates [1].

Surveillance studies have shown high resistance rates among urinary isolates of *Enterococcus*, with resistance to fluoroquinolones and glycopeptides exceeding 40-50%, indicating a troubling escalation in AMR within UTIs [2]. The increasing incidence of vancomycin-resistant and multidrug-resistant (MDR) *Enterococcus* species poses a major clinical challenge. These drug-resistant strains increase mortality rates, hospital stays and the use of healthcare resources, significantly impacting patient outcomes [3-4]. This highlights the need for more effective, region-specific data. These issues are especially concerning in Indian tertiary care settings that serve diverse patient populations. These clinical environments often face unique challenges, including considerable pressure of antimicrobial selection and a higher proportion of vulnerable patients, who include critically ill, catheterised and surgically treated patients, who are at a higher risk of acquiring resistant infections of *Enterococcal* uropathogens. Despite growing concerns about drug resistance, a critical gap exists in the availability of region-specific data, particularly in correlating AMR patterns in *Enterococci* with detailed clinical outcomes, especially within rural and semi-urban Indian healthcare settings. This lack of region-specific data and localised evidence hinders the development of targeted infection control strategies, effective empirical treatment guidelines and strict antimicrobial stewardship programs, leading to suboptimal patient care, extended hospital stay and increased healthcare costs. Studies show that few researchers have thoroughly investigated the association between AMR profiles and their associated clinical outcomes.

The present study aimed to evaluate the AMR patterns and clinical outcomes of uropathogenic *Enterococcus* species, specifically *E. faecalis* and *E. faecium*, isolated from hospitalised patients. The primary objective was to assess the AMR patterns of *Enterococcal* isolates from UTIs. The secondary objectives were to determine the distribution of *E. faecalis* and *E. faecium* across hospital settings and demographic variables (age and sex), and to correlate resistance categories (MDR/extensively drug-resistant (XDR)/pandrug-resistant (PDR)) with clinical and diagnostic parameters, including type of infection, length of hospital stay, Acute Physiology and Chronic Health Evaluation II (APACHE II) score, treatment outcomes and hospital location.

## Materials and methods

Ethical approval and study design

The study received ethical clearance from the Institutional Ethics Committee (IEC) of Krishna Institute of Medical Sciences, Krishna Vishwa Vidyapeeth Deemed to be University, Karad, as indicated by letter no. KIMSDU/IEC/04/2023 and B.K.L. Walawalkar Rural Medical College, letter no. BKLWRMC/IEC/43/2023.

This prospective observational laboratory-based study was conducted in the microbiology department of a tertiary care hospital, with subsequent collection of clinical and demographic data for correlation with AMR patterns.

Study setting and participants

This prospective observational laboratory-based study of two years' duration was conducted in the microbiology department of a tertiary care hospital in southwest Maharashtra from October 2023 to October 2025. All urine specimens obtained from hospitalised patients, including those admitted to the inpatient department (IPD) and intensive care unit (ICU), yielded *Enterococcus* species. Specimens from outpatients, mixed or repeat urine samples, and records with incomplete data were excluded from the study.

Sample size

During the two-year study period, 490 urine specimens were received from IPD patients, including ICU cases, of which 30 isolates (6.12%) yielded *Enterococcus* species. All non-duplicate isolates were included, and no previous sample size calculation was performed. The relatively small sample size reflects the low isolation rate of *Enterococci* in urine samples. The dataset comprised *E. faecalis* (n = 14) and *E. faecium* (n = 16).

Laboratory method

Sample Processing and Species Identification

Midstream clean-catch and catheterised urine specimens were collected in sterile, wide-mouthed containers using standard aseptic precautions. Samples were transported promptly to the microbiology laboratory and processed within one to two hours of collection; if a delay was anticipated, specimens were stored at 2-8°C and processed within 24 hours. Urine samples were cultured using a calibrated loop (0.001 mL) on cysteine-lactose-electrolyte-deficient (CLED) (HiMedia Laboratories Pvt. Ltd., Mumbai, India) and blood agar (HiMedia Laboratories Pvt. Ltd., Mumbai, India) plates and incubated aerobically at 35-37°C for 18-24 hours. Significant bacteriuria was interpreted based on standard colony count criteria. *Enterococcus* species was identified using conventional biochemical methods, and antimicrobial susceptibility testing (AST) was performed according to standard guidelines.

*Enterococcal* isolates were identified using Gram’s staining, bile-esculin hydrolysis, catalase test, growth in 6.5% sodium chloride and arabinose fermentation test for species differentiation (*E. faecalis* vs. *E. faecium*) [5]. American Type Culture Collection (ATCC) *Enterococcus faecalis* 29212 (Manassas, VA, USA) was used for quality control.

Antimicrobial Susceptibility Testing (AST)

AST was performed using the conventional Kirby-Bauer disc diffusion method and interpreted according to the Clinical and Laboratory Standards Institute (CLSI) M100, 33rd edition guidelines [6]. High-level streptomycin (HSL) and high-level gentamicin (HLG) tests were performed as recommended. Minimum inhibitory concentrations (MICs) for vancomycin were determined using the broth microdilution method, following the CLSI M07 guidelines [7]. Antibiotics evaluated were ampicillin 10 µg, penicillin-G 10 U, nitrofurantoin 300 µg, vancomycin (vancomycin hydrochloride potency>950 µg/mL, CMS 217), linezolid 30 µg, ciprofloxacin 5 µg, levofloxacin 5 µg, HLS 300 µg, fosfomycin 200 µg, tetracycline 30 µg, doxycycline 30 µg, and minocycline 30 µg (HiMedia Laboratories Pvt. Ltd., Mumbai, India).

Demographic and clinical data collection

Clinical and demographic data were collected using standardised forms from patients' medical records. Demographic variables, such as hospital location (IPD and ICU), sex (male and female) and age (1-17, 18-35, 36-60, 61-80 and >80 years), were recorded for all patients. These categories were used to compare patient characteristics between *E. faecalis* and *E. faecium* isolates.

Antibiotic-resistance classification

Isolates were categorised into MDR, XDR and PDR and non-MDR classes according to standard international criteria. MDR was defined as non-susceptibility to a minimum of one agent in three or more antimicrobial categories, XDR as non-susceptibility to all but one or two categories, and PDR as non-susceptibility to all agents across all antimicrobial categories. These standardised definitions enable consistent comparisons of resistance patterns across studies [8].

The Antimicrobial Resistance Index (ARI) and Multiple Antibiotic Resistance Index (MARI) were determined using established methods [9]. The ARI was calculated as the average number of antibiotics to which each isolate was resistant (total number of resistance events divided by the total number of isolates). The MARI was calculated for each isolate as the ratio of the number of antibiotics to which the isolate was resistant to the total number of antibiotics tested against that isolate (MARI = a/b) [9].

Length of stay and treatment response

Length of hospital stay (LOS) was calculated for each patient and summarised as the median and interquartile range (IQR). Treatment response was categorised as improved, left against medical advice (LAMA), or death based on clinical documentation and discharge outcomes. These parameters were analysed in relation to *Enterococcal *species and resistance classes.

Diagnosis categories

Clinical diagnostic parameters were grouped into predefined categories, including acute kidney injury (AKI), obstructive uropathy, renal conditions or urosepsis, obstetric conditions, critical illness or septic shock, and other medical or surgical conditions (orthopaedic, haematological, oncological, neurological, geriatric, cardiovascular and respiratory).

Illness severity assessment

Severity of illness was assessed using the APACHE II score, developed by Knaus et al. (1985) [10]. The score comprises 12 physiological variables, including age and chronic health points, and was calculated at patient admission. The APACHE II scores were categorised as follows: low (0-9), moderate (10-19), high (20-29) and severely high (≥30) to evaluate the relationship between severity of illness and resistance patterns in *Enterococcal* isolates. APACHE II scores were calculated at admission based on documented clinical and laboratory parameters. 

Types of urinary tract infections (UTIs)

According to the Indian Council of Medical Research (ICMR) antimicrobial guidelines, an uncomplicated UTI (uUTI) is defined as an infection in healthy, non-pregnant, premenopausal women without structural or functional urinary tract abnormalities and without immunocompromising conditions [11]. In contrast, complicated UTIs (cUTIs) include any urinary infection occurring in the presence of male sex, pregnancy, childhood or elderly age with comorbidities, diabetes, immunosuppression, chronic kidney disease, or anatomical or functional abnormalities, such as urinary tract obstruction, stones, strictures, vesicoureteral reflux, or neurogenic bladder [11-12]. The ICMR guidelines also classify UTIs as complicated when they are device-associated (e.g. catheter-associated UTI, stents, nephrostomy tubes) or healthcare-associated, and when they follow recent hospitalisation or instrumentation [11]. Severe clinical presentations, such as perinephric abscess, urosepsis, and pyelonephritis with systemic features, are also included under the complicated category [11,13].

Statistical analysis

Data were compiled using Microsoft Excel (version 14.0; Redmond, WA, USA) and analysed using Statistical Package for the Social Sciences (SPSS) versions 25.0 and 26.0 (released 2017 and 2018, respectively; IBM Corp., Armonk, NY, USA).

Descriptive statistical methods were used to summarise the variables such as demography, age, sex, and hospital location. Categorical variables, such as species distribution, percentages of AMR, types of infection and treatment response, are expressed as frequencies and proportions. Continuous variables, such as length of hospital stay (LOS), are statistically summarised using the median and inter-quartile range (IQR).

Comparisons between *Enterococcal *species (*E. faecalis* and *E. faecium*), resistance categories (non-MDR, MDR, XDR and PDR), were performed using Fisher’s exact test for categorical variables. Fisher’s exact test was selected due to small cell counts. This method provides valid p-values without requiring the Chi-square test. Due to limitations in the available statistical output, test statistics, degrees of freedom and effect sizes could not be obtained; hence, only p-values are reported.

Statistical significance was considered if p < 0.05. Clinical parameters, such as infection category, LOS, and treatment response, were evaluated in relation to species distribution and drug resistance profiles.

## Results

Demographic characteristics of patients with *Enterococcal* isolates

Table 1 shows that 30 *Enterococcal* isolates were analysed, comprising *E. faecium *(n = 16, 53.33%)* and E.* faecalis (n = 14, 46.67%). The majority of isolates were obtained from the IPD (19, 63.33%), whereas 11 (36.67%) were from the ICU. The distribution of species across hospital locations was not statistically significant (p = 0.47).

The sex distribution was equal, with 15 male (50%) and 15 female (50%) patients, and no significant association was observed between sex and *Enterococcus* species (p = 0.72).

Most infections occurred in the 36-60-year age group (13, 43.33%), followed by the 61-80-year age group (10, 33.33%). Fewer cases were observed in the 18-35-year and > 80-year groups. There was no statistically significant difference in species distribution across age groups (p = 0.43).

**Table 1:** Demographic Characteristics of Patients with Enterococcal Isolates Age distribution reflects the age at admission. *The 1-17 year group was excluded due to zero cases in both species, rendering statistical testing invalid and unnecessary. IPD: inpatient department; ICU: intensive care unit

Category	Subgroup	*E. faecalis* n (%)	*E. faecium* n (%)	Total n (%)	p-value
Hospital location	IPD	10 (71.43)	9 (56.25)	19 (63.33)	0.47
	ICU	4 (28.57)	7 (43.75)	11 (36.67)	
Gender distribution	Male	6 (42.86)	9 (56.25)	15 (50)	0.72
	Female	8 (57.14)	7 (43.75)	15 (50)	
Age group*	18-35 years	4 (28.57)	1 (6.25)	5 (16.67)	0.43
	36-60 years	5 (35.71)	8 (50)	13 (43.33)	
	61-80 years	4 (28.57)	6 (37.5)	10 (33.33)	
	>80 years	1 (7.14)	1 (6.25)	2 (6.67)	
Total	—	14 (46.67)	16 (53.33)	30 (100)	—

Antibiotic resistance profile of *Enterococcus faecalis* and *Enterococcus faecium*

Table 2 presents a wide disparity in the AMR profiles of *E. faecalis *and *E. faecium* isolates. *E. faecium* demonstrated total resistance (16, 100%) to both penicillin and ampicillin, which was significantly higher than the resistance observed in *E. faecalis *(3, 21.4%), indicating strong statistical significance. Moreover, *E. faecium* exhibited 16 (100%) resistance to fluoroquinolones (ciprofloxacin and levofloxacin), exceeding the resistance rate observed in *E. faecalis *(11, 78.5%). Further, the two species were distinguished by their levels of resistance to aminoglycosides, with *E. faecium* showing (15, 93.8%) resistance to gentamicin and 100% resistance to streptomycin, compared with (9, 64.2%) and (4, 28.5%) resistance, respectively, for *E. faecalis*. The most remarkable difference was observed in the response to urinary antibiotics, with *E. faecium* exhibiting (14, 87.5%) resistance to nitrofurantoin, whereas *E. faecalis* was completely susceptible to this antibiotic. Both *Enterococcal* species remained highly susceptible to vancomycin and linezolid, with no significant interspecies difference. A statistically significant difference in fosfomycin resistance was observed between the two species, with *E. faecium *showing 100% resistance compared to 21.4% in *E. faecalis* (p = 0.00002). Resistance to tetracycline group antibiotics was higher in *E. faecalis* than in *E. faecium*, although the difference was not statistically significant (p = 0.573).

**Table 2:** Antibiotic Resistance Profile of Enterococcus faecalis and Enterococcus faecium Fisher’s exact test was used because of the small cell counts. Statistical significance was set at p < 0.05. Counts represent the number of resistant isolates. MIC: minimum inhibitory concentration; HLS: high-level streptomycin; HLG: high-level gentamicin

Antibiotics	*E. faecalis* (n = 14)	*E. faecium* (n = 16)	p-value	Brief inference
Ampicillin (10 µ g)	3 (21.4%)	16 (100%)	0.00002	*E. faecium* shows extremely high resistance; species difference is highly significant
Penicillin-G (10 U)	3 (21.4%)	16 (100%)	0.00002	Markedly higher resistance in *E. faecium*; strong statistical significance
Nitrofurantoin (300 µg)	0 (0%)	14 (87.5%)	6.6E-07	*E. faecium *is* *highly resistant; *E. faecalis* is fully susceptible - very strong significance
Vancomycin (MIC detection)	1 (7.1%)	1 (6.2%)	1	No difference; both species remain largely susceptible
Gentamicin (HLG) (120 µg)	9 (64.2%)	15 (93.8%)	0.041	Higher resistance in *E. faecium*; statistically significant.
Linezolid (30 µg)	1 (7.1%)	2 (12.5%)	0.587	No significant difference; both species are generally susceptible
Ciprofloxacin (5 µg)	11 (78.5%)	16 (100%)	0.044	*E. faecium* shows higher resistance; significant difference
Levofloxacin (5 µg)	11 (78.5%)	16 (100%)	0.044	Similar to ciprofloxacin - significantly higher resistance in *E. faecium*
Streptomycin (HLS) (300 µg)	4 (28.5%)	16 (100%)	0.000093	Very high resistance in *E. faecium* compared to *E. faecalis*
Fosfomycin (200 µg)	3 (21.4%)	16 (100%)	0.00002	Strongly significant higher resistance in* E. faecium*
Tetracycline (30 µg)	13 (92.8%)	9 (56.2%)	0.573	No significant difference; *E. faecalis *shows higher resistance, but not statistically meaningful
Doxycycline (30 µg)	13 (92.8%)	9 (56.2%)	0.573	Not significant
Minocycline (30 µg)	13 (92.8%)	9 (56.2%)	0.573	Not significant

Antibiotic class-wise resistance profile of enterococcal isolates

Table 3 shows the distribution of antibiotic resistance by class. *E. faecium* showed comparatively higher resistance to antibiotics than *E. faecalis.* *E. faecium* exhibited complete resistance, whereas *E. faecalis *showed (3, 21.4%) resistant isolates to β-lactams, including ampicillin and penicillin. The nitrofuran class showed the highest difference, with *E. faecalis* being totally susceptible to nitrofurantoin, as compared to *E. faecium* (14, 87.5%) resistance. *E. faecium* showed higher resistance to aminoglycosides (HLG/HLS) and fluoroquinolones as compared to *E. faecalis*. *E. faecium* showed total resistance to fosfomycin, as compared to (3, 21.4%) in *E. faecalis.* No difference was observed in the resistance of *Enterococcal* isolates to vancomycin and linezolid, and both *Enterococcal* species showed high tetracycline resistance, though not statistically significant. Overall, *E. faecium* demonstrated greater resistance to most of the antibiotic classes.

**Table 3:** Antibiotic Class-Wise Resistance Profile of Enterococcal Isolates Fisher’s exact test was used because of small cell counts. HLG: high-level gentamicin; HLS: high-level streptomycin

Antibiotic class	*E. faecalis *(n = 14)	*E. faecium* (n = 16)	p-value	Brief inference
β-lactams				
Ampicillin	3 (21.4%)	16 (100%)	0.00002	Significantly higher resistance in E. faecium
Penicillin	3 (21.4%)	16 (100%)	0.00002	Markedly higher resistance in E. faecium
Nitrofuran				
Nitrofurantoin	0 (0%)	14 (87.5%)	< 0.000001	E. faecium is highly resistant; E. faecalis is fully susceptible
Glycopeptide				
Vancomycin	1 (7.1%)	1 (6.2%)	1	No significant species difference
Aminoglycosides (high-level)				
Gentamicin (HLG)	9 (64.2%)	15 (93.8%)	0.041	Higher resistance in E. faecium
Streptomycin (HLS)	4 (28.5%)	16 (100%)	0.000093	Very high resistance in E. faecium
Oxazolidinone				
Linezolid	1 (7.1%)	2 (12.5%)	0.587	No significant species difference
Fluoroquinolones				
Ciprofloxacin	11 (78.5%)	16 (100%)	0.044	Higher resistance in E. faecium
Levofloxacin	11 (78.5%)	16 (100%)	0.044	Higher resistance in E. faecium
Phosphonic acid				
Fosfomycin	3 (21.4%)	16 (100%)	0.00002	Significantly higher resistance in E. faecium
Tetracycline class				
Tetracycline	13 (92.8%)	9 (56.2%)	0.573	No significant species difference
Doxycycline	13 (92.8%)	9 (56.2%)	0.573	No significant species difference
Minocycline	13 (92.8%)	9 (56.2%)	0.573	No significant species difference

Antibiotic Resistance Index (ARI) and Multiple Antibiotic Resistance Index (MARI) of *Enterococcal *isolates

Table 4 displays the ARI and MARI of both *Enterococcus* species. *E. faecalis* (n = 14) exhibited a total AMR score of 85, with an ARI of 6.07 and MARI of 0.47, indicating that it has originated from a high-risk source with significant multidrug resistance. In contrast, *E. faecium* (n = 16) demonstrated markedly higher resistance levels, with a total AMR score of 155, an ARI of 9.69 and an MARI of 0.75. These metrics specifically categorise *E. faecium* as a high-risk organism, highlighting its severe multidrug resistance and considerable antibiotic exposure in hospitals.

**Table 4:** Antibiotic Resistance Index (ARI) and Multiple Antibiotic Resistance Index (MARI) of Enterococcal Isolates Antibiotic Resistance Index (ARI) = (total resistance events/(total number of isolates × total number of antibiotics tested)) Multiple Antibiotic Resistance Index (MARI) = (number of antibiotics to which an isolate is resistant/total number of antibiotics tested for that isolate) MARI > 0.2 indicates a high-risk contamination source

Species	ARI	MARI	Interpretation
*E. faecalis* (n = 14)	6.07	0.47	High-risk source, multidrug resistance evident
*E. faecium* (n = 16)	9.69	0.75	Very high-risk; severe multidrug resistance and hospital antibiotic pressure

Antimicrobial resistance patterns and clinical characteristics of enterococcal isolates

Table 5 presents a detailed analysis of 30 *Enterococcal* isolates, which shows that *E. faecalis* (n = 14) exhibited (6, 20%) MDR, (2, 6.67%) XDR, with (6, 20%) classified as non-MDR and no instances of PDR. In contrast, *E. faecium* (n = 16) displayed a higher resistance profile, with (8, 26.67%) MDR, (7, 23.33%) XDR and (1, 3.33%) PDR. Notably, *E. faecium* was associated with all PDR cases and most XDR infections, indicating a more resistant phenotype than that of *E. faecalis.* The overall median length of hospital stay among *Enterococcal* infections was six days (interquartile range (IQR): 3-10 days). Among the subgroups, *E. faecalis* non-MDR isolates had the shortest hospital stay (three days (IQR: two to six days)), whereas *E. faecalis* XDR isolates were associated with longer stays (10 days (range: 7-13 days)). Similarly, *E. faecium* XDR isolates demonstrated prolonged hospitalisation (8 days (IQR: 4-10 days)). MDR isolates of both species showed comparable median durations (*E. faecalis*: 6.5 days (IQR: 3-23 days), *E. faecium*: 6.5 days (IQR: 5-10.5 days)). A single *E. faecium* PDR case had a six-day hospital stay. The most common diagnoses were AKI/obstructive uropathy/renal urosepsis (15, 50%), critical illness/septic shock (five, 16.67%) and conditions related to orthopaedics/oncology/geriatrics (seven, 23.33%). APACHE II severity scores showed that most patients were in the moderate range (10-19) (13, 43.33%), with 12 (40%) cases in the high range (>20), and 2 (6.67%) in the severely high range (>30). Clinical outcomes showed that 23 patients (76.67%) improved, 3 patients (10%) had LAMA, and 4 patients (13.33%) did not survive, mainly in the *E. faecium* MDR/XDR category. The observed mortality represents all-cause in-hospital mortality, and no direct attribution to *Enterococcal* infection was established. Most infections originated in the IPD wards (19, 63.33%) and the ICU (11, 36.67%), indicating a significant burden of infections in critical care. In the present study, the majority of *Enterococcal* infections (29, 96.7%) were classified as cUTI, and one case (1, 3.3%) was identified as a uUTI. Among the cUTI cases, both MDR and non-MDR phenotypes were observed, whereas the uUTI case did not exhibit broad resistance. This distribution highlights a high prevalence of complicated infections among hospitalised patients. The age distribution showed that most patients (n=18, 60%) were aged 36-60 years, followed by those aged 61-80 years (n=10). Due to small subgroup sizes, no statistical comparisons were performed for resistance categories and clinical outcomes; findings are presented descriptively.

**Table 5:** Antimicrobial Resistance Patterns and Clinical Characteristics of Enterococcal Isolates Data are presented as numbers (percentage) or medians (interquartile range (IQR)). For groups with very small sample sizes (n ≤ 2), range or single values are reported instead of IQR. No statistical comparison was performed due to small subgroup sizes; results are presented descriptively. MDR: multidrug-resistant; XDR: extensively drug-resistant; PDR: pandrug-resistant; AKI: acute kidney injury; cUTI: complicated urinary tract infection; uUTI: uncomplicated urinary tract infection; IPD: inpatient department; ICU: intensive care unit; LAMA: left against medical advice; IQR: interquartile range; NA: not applicable

Outcomes parameters	*E. faecalis *MDR (n = 6)	*E. faecalis* XDR (n = 2)	*E. faecalis* non-MDR (n = 6)	*E. faecium* MDR (n = 8)	*E. faecium* XDR (n = 7)	*E. faecium *PDR (n = 1)	Total n (%)/ LOS median (IQR)
Length of stay-median days (IQR days)	6.5 (3-23)	10 (7-13)	3 (2-6)	6.5 (5-10.5)	8 (4-10)	6 (NA)	6 (3-10)
Diagnosis type							
AKI/obstructive uropathy/renal urosepsis	3	1	2	3	5	1	15 (50)
Obstetric conditions	1	0	2	0	0	0	3 (10)
Critical illness/septic shock	1	0	1	1	2	0	5 (16.67)
Other medical/surgical conditions	1	1	1	4	0	0	7 (23.33)
APACHE II category							
Low (0-9)	1	0	2	0	0	0	3 (10)
Moderate (10-19)	3	1	2	4	3	0	13 (43.33)
High (20-29)	2	1	2	3	3	1	12 (40)
Severely high (≥30)	0	0	0	1	1	0	2 (6.67)
Treatment response							
Improved	6	2	4	5	6	0	23 (76.67)
LAMA	0	0	2	0	1	0	3 (10)
Death	0	0	0	2	1	1	4 (13.33)
Hospital location							
IPD	4	2	4	4	4	1	19 (63.33)
ICU	2	0	2	4	3	0	11(36.67)
Type of UTI							
cUTI	6	2	6	7	7	1	29 (96.67)
uUTI	0	0	0	1	0	0	1 (3.33)
Age group							
18-35 years	1	0	0	0	0	0	1 (3.33)
36-60 years	4	1	4	6	3	0	18 (60)
61-80 years	1	1	2	1	4	1	10 (33.33)
>80 years	0	0	0	1	0	0	1 (3.33)

## Discussion

*Enterococcus* species constituted 30 (6.12%) of culture-positive urine samples from inpatients, with *E. faecalis* (14, 46.7%) and *E. faecium *(16, 53.3%) being the most common uropathogens. This aligns with previous studies, in which *Enterococcus* comprised 4-12% of urine culture isolates among hospitalised patients [14]. Yadav et al. reported a 7.1% rate of *Enterococcal i*solation in a North Indian tertiary care setting [15]. *E. faecalis *and *E. faecium* accounted for 46.7% and 53.3% of isolates, showing a slight predominance of *E. faecium. *This reflects the increasing proportion of *E. faecium* infection in India. Yadav et al. reported similar results, with 50.3% *E. faecalis *and 47.5% *E. faecium* [15]. *E. faecium *was more frequently isolated from ICU samples, although statistically non-significant (p = 0.47), consistent with other studies showing increased *E. faecium,* especially in high-dependency units [15]. Sex-wise distribution showed no significant association (p = 0.72), consistent with previous studies [3]. A statistically non-significant trend toward higher *E. faecium* isolation among older adults (p = 0.43) is consistent with scientific evidence that age-related vulnerability may reflect existing comorbidities [16].

Recent research findings suggest that *E. faecium* exhibits significantly higher resistance levels than *E. faecalis,* particularly to ampicillin, penicillin and fluoroquinolones. For example, *E. faecium* exhibited total resistance to both ampicillin and penicillin, whereas *E. faecalis* demonstrated a resistance rate of 21.4% (p<0.001). Similarly, resistance to fluoroquinolones was comparatively greater in *E. faecium* (100%) than in *E. faecalis* (78.5%). The same pattern was observed for aminoglycosides such as gentamicin and streptomycin, with *E. faecium* showing resistance rates of 93.8% and 100%, respectively, compared with 64.2% and 28.5% in *E. faecalis*. The most remarkable difference was observed with nitrofurantoin, in which *E. faecalis *remained fully susceptible, whereas *E. faecium* exhibited 87.5% resistance (p < 0.05). Resistance to tetracycline group antibiotics was higher in *E. faecalis* compared to *E. faecium*, although this difference did not reach statistical significance (p = 0.573). Notably, this pattern indicates that tetracycline resistance may follow a species-specific mechanism rather than a uniform resistance trend. From a clinical perspective, the high resistance rates in both species limit the reliability of tetracyclines for empirical therapy. Both *Enterococcal* species demonstrated high susceptibility to vancomycin and linezolid, with no significant difference between them. These findings are similar to those of previous studies that underscore a greater tendency toward multidrug resistance of *E. faecium*, complicating patient treatment and highlighting the necessity for ongoing monitoring [17]. 

Fosfomycin is an established oral agent for uUTIs due to its high urinary concentrations and convenient dosing [14]. In the present study, a significant interspecies difference was observed: *E. faecium* demonstrated complete resistance, whereas *E. faecalis* showed low resistance. These findings suggest that fosfomycin may have limited utility against *E. faecium*, whereas it may remain a useful option for *E. faecalis* infections. Our findings likely reflect local antimicrobial pressure and hospital epidemiology, and we have interpreted them cautiously without overgeneralisation.

Table 4 shows the AMR patterns of *E. faecalis *(n = 14) and *E. faecium* (n = 16), which demonstrates significant AMR, with *E. faecium* showing higher levels of resistance (total resistance: 155, ARI: 9.69 and MARI: 0.75) as compared to *E. faecalis* (total resistance: 85, ARI: 6.07 and MARI: 0.47). This finding suggests that *E. faecium* is a high-risk species with severe multidrug resistance, which is influenced by antibiotic use in hospitals, whereas *E. faecalis* remains a high-risk source of multidrug resistance. The increased resistance in *E. faecium* aligns with global surveillance findings reporting growing resistance to drugs such as aminoglycosides, fluoroquinolones and tetracyclines, driven by genes such as aac(6')-Ie-aph(2")-Ia and tetM/tetL [18,19].

Table 5 presents AMR patterns, indicating that *E. faecalis* (n = 14) exhibited 42.8% MDR, 14.2% XDR, 0% PDR, and 42.8% non-MDR. In contrast, *E. faecium* (n = 16) showed higher drug resistance, with 50% MDR, 43.7% XDR and 6.2% PDR isolates, accounting for all PDR cases and the majority of XDR infections, highlighting its comparatively greater resistance [20]. The median hospital stay of six days (IQR: 3-10) indicates a generally moderate duration of hospitalisation among *Enterococcal* infections, with variability by resistance profile. Non-MDR *E. faecalis* cases had the shortest stays. In contrast, a trend toward longer hospital stay was observed among patients with XDR isolates, suggesting greater disease severity and/or treatment challenges; however, this finding should be interpreted cautiously due to small subgroup sizes. These findings highlight the clinical impact of AMR on hospital LOS, which aligns with recent studies linking MDR/XDR *Enterococcus* to extended hospitalisation [20]. Most infections were cUTIs (96.7%), with only one case of uUTI. cUTI cases included both MDR and non-MDR types, whereas the uUTI case showed no significant resistance. This finding aligns with global patterns indicating a high proportion of cUTI among hospitalised patients, particularly those with resistant *Enterococcus *strains [20]. A study of clinical outcomes indicated that most patients showed improvement (23/30). Still, mortality was more frequent among patients with MDR/XDR *E. faecium*; however, a direct causal association cannot be established. They were strongly associated with cUTI, especially when complicated by sepsis, septic shock or other severe comorbidities, highlighting the risk of high mortality in complicated *Enterococcus* UTIs [21].

Empirical therapy in the context of ICMR guidelines

In the present study, the high resistance observed to fluoroquinolones, high-level aminoglycosides, and fosfomycin underscores the limited role of these agents in empirical therapy. These findings are consistent with the recommendations of the ICMR AMR guidelines, which discourage the empirical use of these drugs for Enterococcal infections due to rising resistance patterns [12]. The detection of vancomycin-resistant *Enterococci *(VRE) in both species further emphasises the need for species-specific, susceptibility-guided therapy.

Limitations

This study has several limitations, including the absence of a priori sample size calculation, a relatively small sample size and a single-centre design, which may limit generalisability. The lack of molecular testing precluded detailed characterisation of resistance mechanisms. Information on previous urinary catheterisation was unavailable, preventing the assessment of catheter-associated infections. Additionally, the small sample size and very small subgroup numbers (e.g. PDR (n = 1), XDR (n = 2-7)) limited statistical power; consequently, analyses relied on Fisher’s exact test, restricting robust estimation of effect sizes and confidence intervals. Subgroup findings should therefore be interpreted with caution.

## Conclusions

*Enterococcus* species are important pathogens in UTIs, particularly among hospitalised patients, with *E. faecalis* and *E. faecium* occurring in comparable proportions. Although patient demographics and baseline clinical characteristics did not significantly influence species distribution, notable differences were observed in AMR patterns. *E. faecium* exhibited higher levels of resistance, including a greater tendency toward MDR and XDR phenotypes, whereas *E. faecalis* generally showed lower resistance; however, these observations should be interpreted cautiously, given the small sample size and subgroup distribution.

A trend toward more severe clinical presentation and longer hospital stay was observed in patients with resistant isolates; however, these findings should be interpreted cautiously and do not establish a direct causal relationship. Adverse outcomes, including mortality, were more frequently observed among patients with highly resistant *Enterococcal* infections, particularly in those with renal impairment and multiple comorbidities, although causality cannot be inferred. These findings underscore the importance of species-level identification, local antimicrobial susceptibility surveillance and strengthened antimicrobial stewardship practices in tertiary care settings. A key strength of this study is the comprehensive antimicrobial profiling of *Enterococcal *isolates across multiple antibiotic classes.
